# Clinico-pathologic Evaluation of Patients with Hemangioendothelioma: A 10-year Experience from Shiraz, Southern of Iran: Short title: Clinicopathologic evaluation of hemangioendothelioma

**DOI:** 10.31661/gmj.v15i.3875

**Published:** 2026-08-16

**Authors:** Mohammad Hossein Anbardar, Mohammad Ali Rahmani, Ali Ameli, Sholeh Afshinpoor, Hamid Zaferani Arani, Nastaran Fooladivanda

**Affiliations:** ^1^ Department of Pathology, Shiraz University of Medical Sciences, Shiraz, Iran; ^2^ Department of Surgery, Shiraz University of Medical Sciences, Shiraz, Iran

**Keywords:** Hemangioendothelioma, Liver, Spleen, Lung, Pathology, Clinical, Tumor

## Abstract

**Background:**

Hemangioendothelioma (HE) represents a group of rare, borderline malignant vascular tumors with diverse clinical presentations and diagnostic challenges. This study from Southern Iran aims to investigate the demographic, clinical, radiological data, histological findings, treatment modalities, survival rates, and other key characteristics of HE cases in both pediatric and adult patients.

**Materials and Methods:**

This is a case series cross-sectional study conducted at Namazi Teaching Hospital and Abuali Sina Hospital, Shiraz University of Medical Sciences. The study involved a retrospective review of medical records from 2009 to 2019, focusing on 22 patients diagnosed with HE. Data were meticulously collected using a standardized collection form. For precise histological examination, tissue slides were extracted and analyzed, incorporating pathological variables and immunohistochemical markers.

**Results:**

In this study, a total of 22 patients diagnosed with HE was examined. Their mean age was 40.6 years with male-to-female ratio of 12:10. The majority of cases in this study were diagnosed with hepatic epithelioid HE (59%). The most commonly reported symptoms included abdominal pain and palpable masses. Radiologically, the tumors primarily appeared as hypo-dense lesions, often accompanied by peripheral rim enhancement. Pathologically, the features such as intracytoplasmic vacuolization, eosinophilic cytoplasm, and specific markers (CD31, CD34) were crucial in accurate diagnosis. Importantly, the study also revealed a positive outcome in terms of patient survival, with only 10% of surgically treated patients succumbing to the disease.

**Conclusion:**

This study provides a detailed clinico-pathologic evaluation of HE from Southern Iran, underscoring the critical relationship between tumor location and clinical presentation. The research highlights that HE, though rare, can occur in pediatric and adults and must be included in the differential diagnosis of vascular lesions across all age groups. From a therapeutic perspective, surgical intervention emerged as the most effective treatment modality, associated with a notably high survival rate of 90% among operated patients.

## Introduction

The term hemangioendothelioma (HE) encompasses various vascular proliferations that can affect the skin, bones, and soft tissues [1]. HE was first described by Enzinger and Weiss in 1982. Their work emphasized that HE should be categorized as neoplasms with a borderline status, representing a transitional point between benign hemangiomas and malignant angiosarcomas [2]. HE encompasses a diverse group of histologically distinct tumors, which include papillary intra-lymphatic HE (also known as Debska tumor), retiform HE, myoid HE, kaposiform HE, epithelioid HE, spindle cell HE, and composite HE [3]. Clinical manifestations of HE is diverse and contingent upon the tumor’s location. Some patients may remain asymptomatic, with the mass being discovered incidentally. However, symptomatic patients can exhibit a wide range of symptoms, from bone pain to neurological issues and systemic manifestations like fatigue, weakness, and weight loss. The severity of symptoms can be influenced by the affected organ or the presence of metastases, leading to respiratory symptoms, pleural effusion, and hemoptysis in cases of lung involvement, or complications such as liver failure, Budd-Chiari syndrome, or portal hypertension in liver involvement. Additionally, some studies have reported pathologic fractures in cases of bone metastasis [4, 5, 6]. HEs are considered low-grade vascular neoplasms with a relatively low metastatic potential, typically exhibiting a tendency to spread locally. Diagnosing HE can be challenging due to its wide range of differential diagnoses. This includes autoimmune diseases like granulomatosis with polyangiitis, infectious diseases such as sarcoidosis, and other malignancies like angiosarcomas and metastatic lesions. Proper diagnosis and differentiation are crucial for determining the appropriate treatment approach and prognosis [4, 7, 8]. HE is an exceptionally rare tumor, and its precise diagnosis can be a formidable challenge, involving clinical, radiological, and histological assessments. Radiological findings from computed tomography (CT) scans and magnetic resonance imaging (MRI) can provide valuable diagnostic clues. However, the gold standard for diagnosing HE relies on a thorough examination of tissue samples, incorporating unique histological, immunohistochemical (IHC), and molecular characteristics [9, 10]. Due to the tumor’s rarity, determining the most effective treatment approach and overall prognosis has remained uncertain. Treatment options span a wide spectrum, from observation for localized disease to surgical resection. Chemotherapy may be considered for cases with multi-organ involvement or metastatic disease. Generally, patients with metastases face a more complex prognosis compared to those with localized disease [11, 12, 13].

### Objectives

This study aimed to extract and evaluate the characteristics of patients with HE tumors. The results of this study will be helpful for clinical diagnosis and decision making of clinicians, pathologists, and surgeons for reducing misdiagnosis and mismanagement of this rare tumor.

## Materials and Methods

### Study design, setting, and participants

This study is a case series cross-sectional analysis conducted at Namazi Teaching Hospital and Abu-Ali Sina Hospital, both affiliated with Shiraz University of Medical Sciences in Shiraz, Fars, Iran. The research involved a retrospective review of medical records spanning from 2009 to 2019, focusing on 22 patients diagnosed with HE. The selection of records was based on specific criteria: patients with a confirmed diagnosis of HE through methods like needle biopsy, open surgical biopsy, or total resection biopsy. Patients with alternative diagnoses, such as angiosarcoma, were excluded from the study. Ultimately, the study comprised 22 eligible patients. This study was approved by the ethical committee at Shiraz University of Medical Science (IR.SUMS.MED.REC.1399.467) and conforms to the principles of the Declaration of Helsinki. All patients have expressed their consent in writing.

### Data collection

All data were collected using a collection form containing the patient’s unit number, age, gender, and pathologic diagnosis, the chief complaint in presentation, past medical disease, imaging studies, lab data, treatment method, survival, and recurrence. Tissue slides were extracted from the data source of the Department of pathology of Shiraz University of Medical sciences. Histologic sections were stained with hematoxylin and eosin technique. IHC studies were performed on formalin fixed, paraffin embedded tissue using the avidin-biotin-peroxidase techniques. Monoclonal antibodies against endothelial markers used include CD31, CD34, and polyclonal antibodies such as factor Ⅷ antigen. Other factors including Ki-67 antibody, anti-SMA (smooth muscle actin) antibody, cytokeratin (CK) AE1-AE3 Cocktail Antibody were used. All source was from VITRO (Master Diagnóstica), Granada, Spain. IHC results report based on the percent of microscopic field staining, as negative for <5%, focally positive for 5-20%, and diffuse positive for >20%. Ki-67 antigen expression reported by percent. Also, it could be quantitated as negligible for <5%, low for 5-10%, moderate for 11-40%, and high for >40%. Pathological parameters were studied in binary (Yes-No model), Numbered (to each subgroup) and semi-quantitatively in random fields and average use. For semi-quantitatively model, the score 1 for low, 2 for medium and 3 for high were considered. Binary model includes pleomorphism, necrosis, eosinophilic cytoplasm, intracytoplasmic vacuolization, irregular nuclei border, coarse chromatin, multiple nucleoli, vascular channeling, glumeruloid formation, tumor calcification, micro-thrombus, spindling and atypical mitosis. Semi-quantitative was used for rate of anaplasia, sclerosis. The average mitotic count per 10 high-power fields (HPF), using a ×40 objective and ×10 ocular (field area = 0.159 mm) was counted for each case.

### Statistical analysis

For the descriptive data analysis, we employed various statistical measures including frequency (N), percentage (%), and the mean with standard deviation. Kolmogorov–Smirnov test is used to assess the normality of the data distribution. The calculation of overall survival time was based on the period from the date of pathological tumor confirmation to either the date of death or the date of the last follow-up. To assess relationships among categorical variables, we utilized the Chi-square test. Statistical significance was determined by p-values, with values less than 0.05 considered statistically significant. All statistical analyses were conducted using SPSS Version 23 (Statistical Package for Social Science). We obtained an ethical approval letter from the Ethical Committee of the Shiraz University of Medical Sciences, Shiraz, Iran.

## Results

**Table:** Table1. Frequency and organ distribution of HE patients.

Type	Frequency	Percent	Male-to-female		Site		
				Liver	Spleen	Lung	Retroperitoneum
Epithelioid HE	17	77.3	8:9	13	0	3	1
Myoid HE	4	18.2	3:1	0	4	0	0
Kaposiform HE	1	4.5	1:0	0	1	0	0
Total	22	100.0	12:10	13	5	3	1

**Table:** Table2. Radiologic findings of all cases with HE.

Patient number	Tumor type	Modality	Number and size of tumor	Site of tumor involvement	Description /Contrast enhancement in CT scan/MRI	Description/Echogenicity in sonography
1	Epithelioid	Sonography	Multiple; <5cm	Liver; Diffuse in parenchyma	N/A	Target-like lesions
2	Epithelioid	N/A	N/A	Liver	N/A	N/A
3	Epithelioid	CT/Sonography	Multiple; >5cm	Liver; Both lobe: first one in segment 6 and 7 in Rt lobe and the second one on lateral segment of Lt lob	Well defined /Both are hypo-dense/ Heterogonous partial enhancement in venous phase and significantly enhancement in delayed phase.	Inhomogeneous hypoechoic mass
4	Epithelioid	MRI/Sonography	Multiple; N/A	Liver; Both lobe: Mostly involve Rt lobe	some having target appearance/ in post gadolinium scan lesions display enhancement	Hypoechoic mass lesion
5	Epithelioid	CT/Sonography	Single; >5cm	Liver; Rt lobe	Hypo-dense with ringed enhancement	N/A
6	Epithelioid	CT/Sonography	Multiple; >5cm	Liver; Rt lobe	Hypo-dense lesions	target lesions with hypoechoic rim and hyperechoic center
7	Epithelioid	N/A	N/A	liver	N/A	N/A
8	Epithelioid	N/A	N/A	liver	N/A	N/A
9	Epithelioid	CT/Sonography	Single; > 5cm	Liver; posterior aspect of the Rt lobe	Hypo-dense	Hypo-echoic with focal ischemia
10	Epithelioid	N/A	N/A	liver	N/A	N/A
11	Epithelioid	N/A	N/A	liver	N/A	N/A
12	Epithelioid	sonography	Multiple; N/A	liver	N/A	Hypo-echoic lesions with heterogeneous pattern
13	Epithelioid	N/A	N/A	liver	N/A	N/A
14	Myoid	N/A	N/A	spleen	N/A	N/A
15	Myoid	CT/Sonography	Single; <5cm	Spleen; Medial aspect of lower pole of spleen	Enhancing but iso-dense to spleen in delay phase	Mixed echogenicity
16	Myoid	CT/Sonography	Single;< 5 cm	Spleen; inferior aspect of spleen	Enhanced in arterial phase but not in venous phase	Irregular/ heterogonous hypoechoic mass
17	Myoid	Sonography	Multiple; <5 cm	Spleen; Diffuse in parenchyma	N/A	Ill-defined and Hypo-echoic
18	Kaposiform	CT/Sonography	Single; < 5 cm	Spleen; upper part of the spleen	Hypo-dense appearance in the arterial and Porto-venous phase	well defined /slightly heterogonous hypoechoic
19	Epithelioid	CT	Single; <5cm	Lung; left lower lobe	is pulmonary infiltration and consolidation/Also have enlarged lymph nodes	N/A
20	Epithelioid	N/A	N/A	lung	N/A	N/A
21	Epithelioid	N/A	N/A	long	N/A	N/A
22	Epithelioid	N/A	N/A	retroperitoneum	N/A	N/A

** N/A:** not available, ** CT:** computed tomography, ** MRI:** magnetic resonance imaging

**Table:** Table3. Lab data, type of specimen, treatment and outcome in patients with HE.

Patient number	Site of tumor	Tumor type	Hb	Platelet	AST	ALT	ALP	Specimen collection	Size; greatest diameter (cm)	Treatment or surgery	Outcome
1	liver	Epithelioid	11.8	227	26	34	240	biopsy	N/A	N/A	9 month, dead
2	liver	Epithelioid	N/A	N/A	N/A	N/A	N/A	biopsy	N/A	Chemotherapy	8 month, dead
3	liver	Epithelioid	12.7	219	26	15	176	resection	5	Liver Transplant	7 years, alive disease free
4	liver	Epithelioid	15	386	21	14	200	resection	7.5	Liver Transplant	7 years, alive disease free
5	liver	Epithelioid	10.6	177	69	13	599	biopsy	N/A	Chemotherapy	1 month, dead
6	Liver	Epithelioid	9.2	251	172	60	3115	biopsy	N/A	N/A	N/A
7	liver	Epithelioid	N/A	N/A	N/A	N/A	N/A	biopsy	N/A	Liver angiography, radio-ablation	9.5 years, alive disease free
8	liver	Epithelioid	N/A	N/A	N/A	N/A	N/A	biopsy	N/A		N/A
9	liver	Epithelioid	N/A	336	16	11	207	resection	3.5	Liver Transplant	10 years, alive disease free
10	liver	Epithelioid	N/A	N/A	N/A	N/A	N/A	biopsy	N/A	N/A	1 month, dead
11	liver	Epithelioid	9.3	100	224	262	144	resection	3	Right lobectomy	2 years; alive disease free
12	liver	Epithelioid	10	343	N/A	N/A	N/A	resection	20	Liver transplant	6 month; alive disease free
13	liver	Epithelioid	N/A	N/A	N/A	N/A	N/A	biopsy	N/A	N/A	2 years; alive disease free
14	spleen	Myoid	13.3	141	28	20	293	resection	N/A	Splenectomy	6 years, alive disease free
15	spleen	Myoid	12.9	273	28	20	293	resection	3	Splenectomy and chemotherapy	6 years, alive disease free
16	spleen	Myoid	12.8	186	11	9	229	resection	3	Splenectomy	7 years, alive with metastasis to liver and lung
17	spleen	Myoid	7.6	118	13	26	276	resection	3	Splenectomy	5 years, alive disease free
18	spleen	Kaposiform	14.6	138	20	33	155	resection	N/A	N/A	N/A
19	lung	Epithelioid	11.4	472	32	15	197	resection	2	N/A	N/A
20	lung	Epithelioid	N/A	N/A	N/A	N/A	N/A	resection	2	N/A	N/A
21	lung	Epithelioid	12.9	294	N/A	N/A	N/A	resection	4	Lung lower lobe resection, 2 session chemotherapy	1 year, alive with metastasis to ribs
22	retroperitoneum	Epithelioid	N/A	N/A	N/A	N/A	N/A	resection	15	N/A	N/A

**N/A:** not available, **Hb:** hemoglobin, **AST:** aspartate aminotransferase, **ALT:** alanine aminotransferase, **ALP:** alkaline phosphatase

**Table:** Table4. Histopathological and IHC findings of patients with HE.

Patients number	Tumor type	Anaplasia	Sclerosis	Non-tumoral feature	Tumor border	Mitotic count (No./10 HPF)	Atypical mitosis	CD31	CD34	Factor VIII	Ki67	CK	SMA
1	Epithelioid	moderate	severe	normal	WD	0	0	Dif +	Foc +	Foc +	3% (negligible)	N	N/A
2	Epithelioid	mild	severe	N/A	N/A	0	0	Dif +	N/A	N/A	N/A	N	N/A
3	Epithelioid	moderate	severe	normal	ID	2	0	Dif +	Dif +	Dif +	2% (negligible)	Foc +	N/A
4	Epithelioid	moderate	severe	normal	ID	2	0	N/A	N/A	N/A	N/A	N/A	N/A
5	Epithelioid	severe	mild	steatosis	WD	2	0	Foc +	N	Dif +	50% (High)	Dif +	N/A
6	Epithelioid	severe	mild	normal	WD	6	P	N/A	N/A	N/A	N/A	N/A	N/A
7	Epithelioid	moderate	severe	N/A	N/A	1	0	Dif +	Dif +	Dif +	5% (Low)	Dif +	N/A
8	Epithelioid	0	moderate	normal	WD	0	0	N/A	N/A	N/A	N/A	N/A	N/A
9	Epithelioid	severe	severe	normal	WD	1	0	N/A	N/A	N/A	N/A	N/A	N/A
10	Epithelioid	moderate	mild	normal	WD	2	0	Dif +	Dif +	N/A	N/A	N/A	N/A
11	Epithelioid	severe	severe	normal	WD	0	0	Dif +	Dif +	Dif +	1% (negligible)	Foc+	N/A
12	Epithelioid	severe	severe	normal	ID	0	0	Dif+	Dif +	Dif +	10% (Low)	N	N/A
13	Epithelioid	moderate	severe	normal	WD	0	0	Dif +	Dif +	Dif +	5% (Low)	N	N/A
14	Myoid	mild	mild	normal	WD	0	0	Dif+	Dif+	Dif+	3% (negligible)	Dif+	N/A
15	Myoid	moderate	moderate	normal	WD	0	0	Dif +	N	Dif+	10%(Low)	N	Dif+
16	Myoid	mild	moderate	normal	WD	0	0	Foc+	Dif+	Dif+	10%(Low)	N	Dif+
17	Myoid	moderate	moderate	normal	WD	2	0	Dif+	N	Dif+	15%(moderat)	N	Dif+
18	Kaposiform	mild	severe	normal	WD	0	0	Dif +	Dif +	Dif +	5% (Low)	N	Dif+
19	Epithelioid	moderate	moderate	normal	WD	3	0	Dif +	N	N	15% (Moderate)	N	N/A
20	Epithelioid	severe	mild	normal	WD	13	0	N/A	N/A	N/A	N/A	N/A	N/A
21	Epithelioid	severe	severe	normal	WD	10	P	Dif +	Dif +	N	70% (High)	Dif +	N/A
22	Epithelioid	moderate	moderate	N/A	WD	0	0	N/A	N/A	N/A	N/A	N/A	N/A

**HPF:** high-power fields, **CK:** cytokeratin, **SMA:** smooth muscle actin, **N/A:** not available, **WD:** well-defined, **ID:** ill-defined, **Dif:** diffusely, **Foc:** focally, **P:** positive, **N:** negative

**Figure-1:**
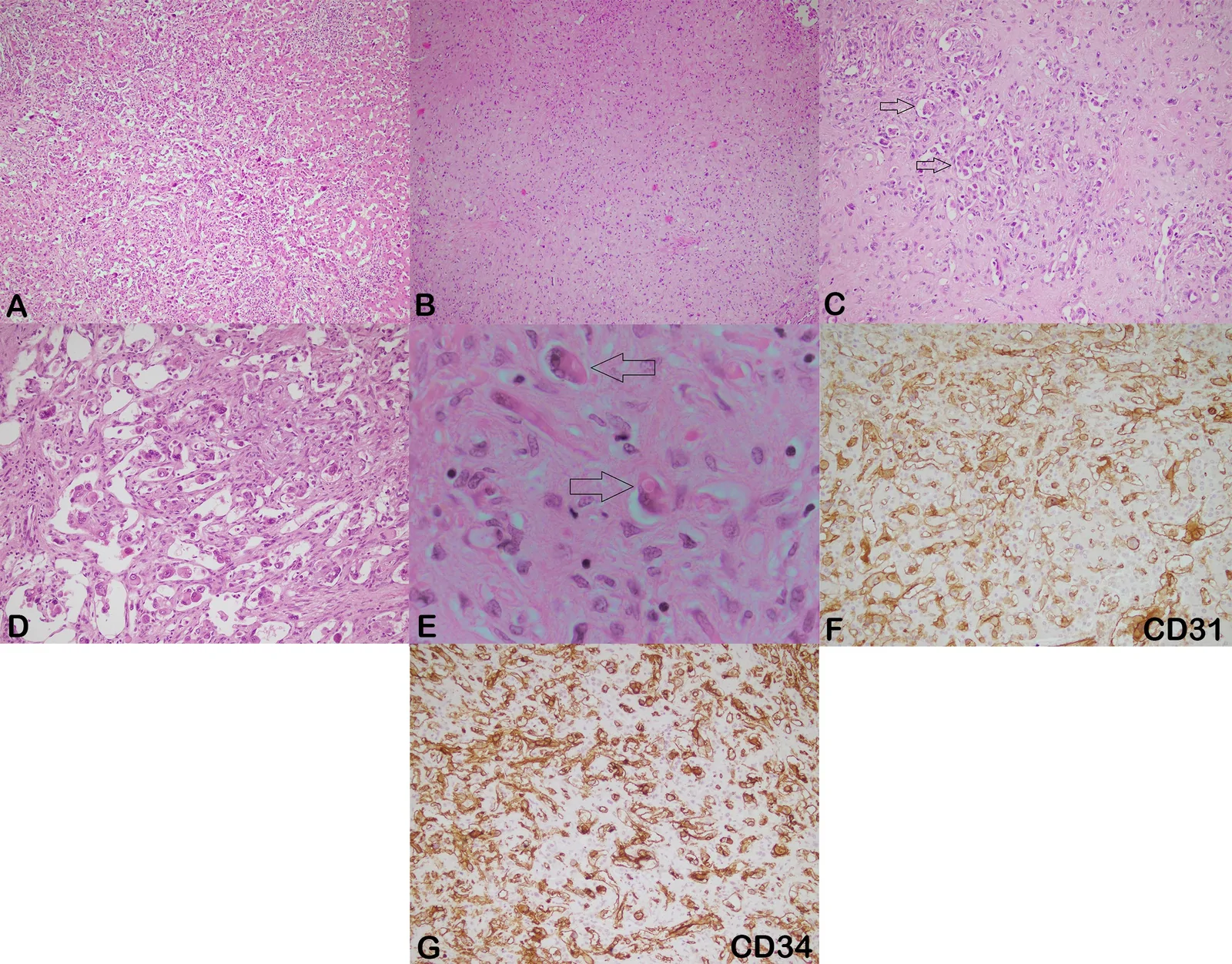
Figure 1. Microscopy and IHC of hepatic epithelioid HE. A) low-power view shows sheets and single cells with invasion into sinusoids, portal and hepatic veins at tumor edge (Hematoxylin and eosin, 100×). B) infiltrative tumor cells in myxo-hyaline and fibrous stroma (Hematoxylin and eosin, 100×). C) arrows show glomeruloid pattern formation (Hematoxylin and eosin, 200×). D) epithelioid cells with eosinophilic cytoplasm, nuclear pleomorphism and prominent nucleoli (Hematoxylin and eosin, 200×). E) arrows show occasional intracytoplasmic vacuolization (Hematoxylin and eosin, 1000×). F) immunoreactivity for CD31 (200×). G) immunoreactivity for CD34 (200×).

**Figure-2:**
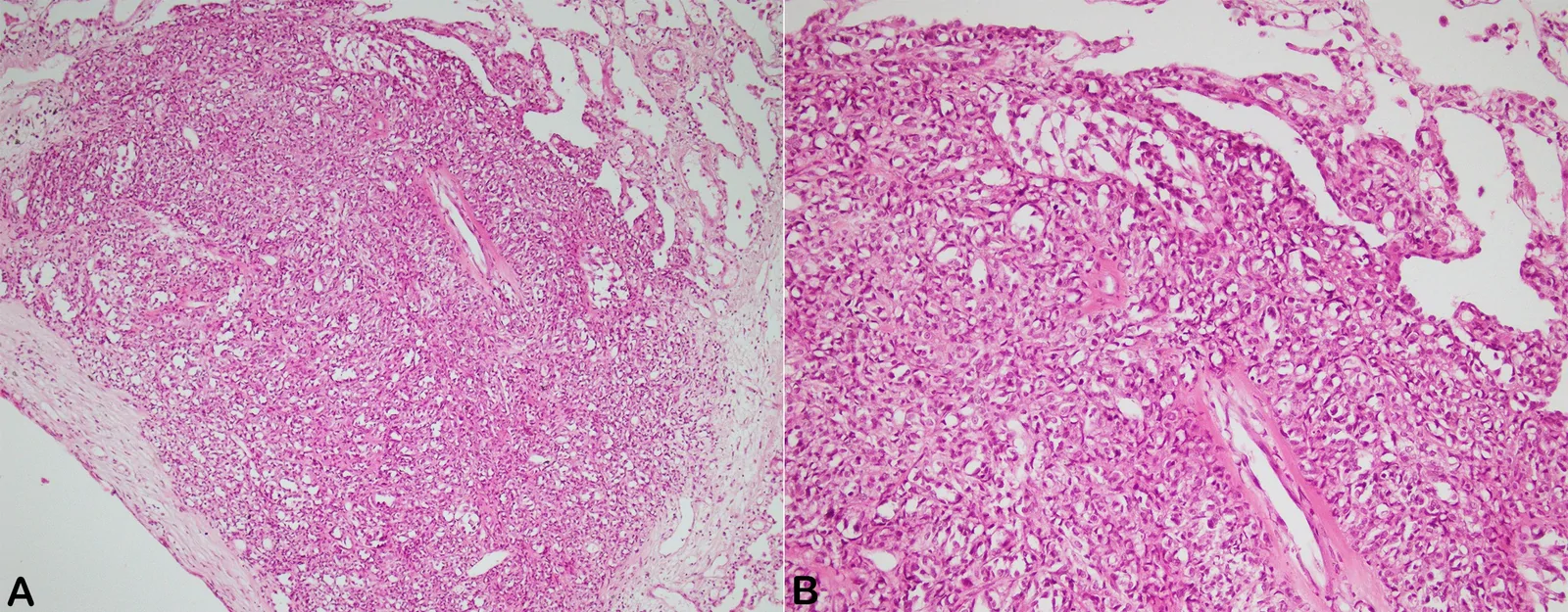
Figure 2. A and B) low-power and high-power view of lung epithelioid HE show solid sheet of epithelioid cells with bland looking appearance (Hematoxylin and eosin, A: 100× and B: 200×).

**Figure-3:**
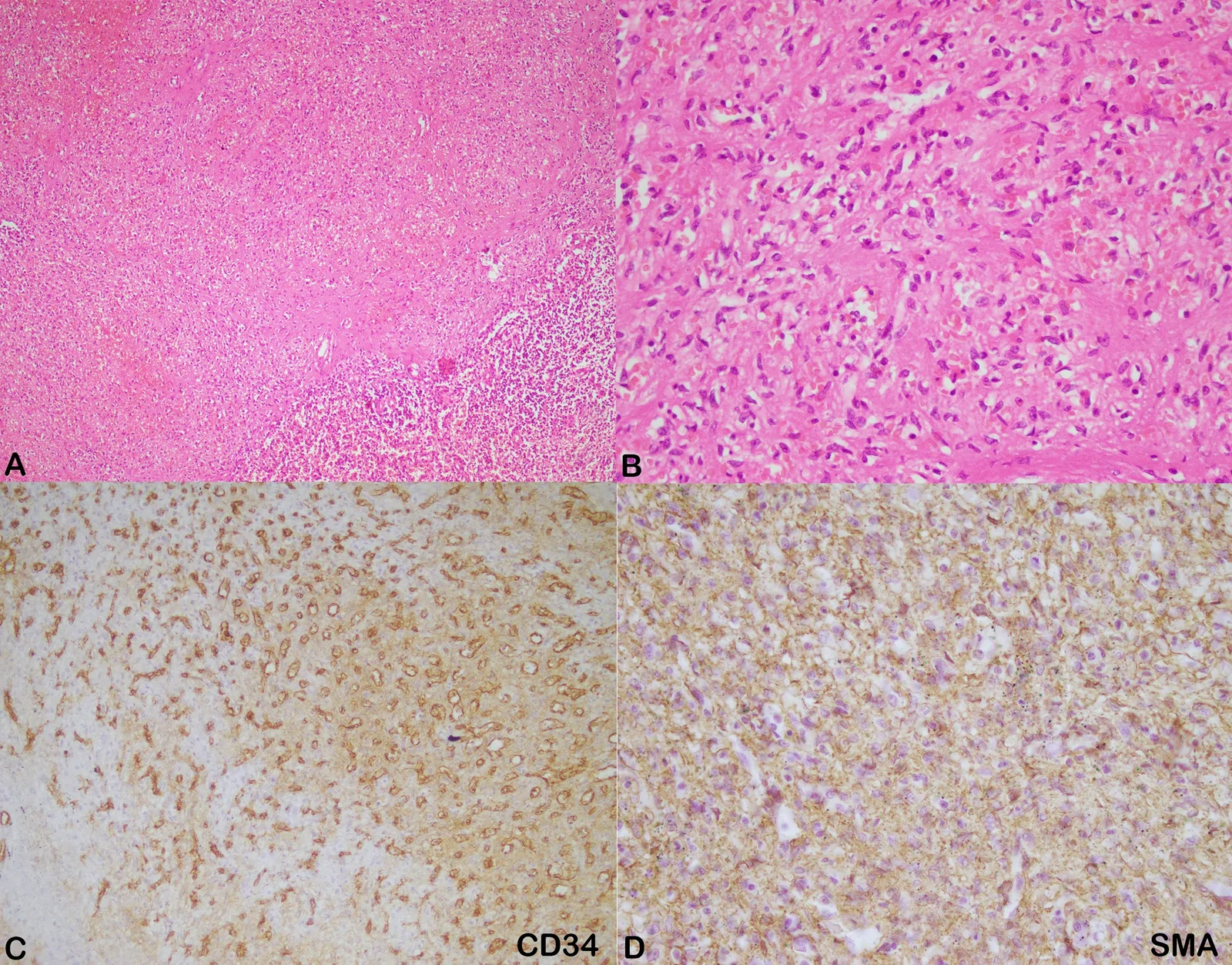
Figure 3. A and B) low-power and high-power view of splenic myoid HE show mesenchymal tumor composed of spindle to histiocytic tumoral cells with small capillary-like blood vessels embedded in eosinophilic stroma (Hematoxylin and eosin, A: 100× and B: 400×). C) immunoreactivity for CD34 (200×). D) immunoreactivity for SMA (400×).

### Demographic and clinical data

In this study, a total of 22 patients diagnosed with HE was examined. Their mean age was 40.6 years, with ages ranging from 2 to 80 years. Among the patients, 54.5% were male, resulting in a male-to-female ratio of 12:10 (Table1). The majority of cases in this study were diagnosed with epithelioid HE (77.3%), followed by myoid HE (18.2%), and kaposiform HE (4.5%). The study did not find any statistically significant relationship between gender and the type of HE (P: 0.388). Additionally, there were no statistically significant associations observed between tumor location, age, and gender (P=0.977 and P=0.299, respectively). In patients with liver tumors caused by HE, clinical presentations often included specific symptoms such as general abdominal pain or right upper abdominal pain, which was the most frequently reported symptom, affecting approximately 50% of all adult patients. Other symptoms included weakness, weight loss, jaundice, and incidental findings. Spleen involvement, mainly associated with myoid type HE, was identified in five patients. The most common presentations in these cases were constitutional symptoms, with generalized weakness being observed in three patients. Two cases were incidentally discovered through routine evaluations. Three cases had lung involvement, and their symptoms included respiratory issues like dyspnea and cough. Some patients experienced more severe symptoms, including hemoptysis and pleuritic chest pain (Table2). Table3 reveals that anemia was the most prevalent laboratory finding among all cases. Among the patients who underwent surgical treatment, an impressive 90% were censored, marking a noteworthy achievement compared to the other treatment groups.

### Pathological findings of epithelioid HE

A comprehensive review of numbered and semi-quantitative parameters was conducted for all 17 cases of epithelioid HE. The findings revealed that among these cases, seven (41.1%) exhibited severe anaplasias, and eight (47%) showed moderate anaplasia. Furthermore, the majority of cases had a high sclerosis index, with ten (58.8%) cases classified as severe (41.1%) and three (17.6%) as having moderate sclerosis. Common histopathological characteristics included intracytoplasmic vacuolization, eosinophilic cytoplasm, and sclerotic stroma. The majority of cases also displayed pleomorphism, coarse chromatin, irregular nuclear borders, prominent nucleoli, glomeruloid formation, and nuclear spindling. Two (11.7%) of the epithelioid cases, involving the lungs, exhibited necrosis upon microscopic evaluation. Micro-thrombus, vascular channels, and multiple nucleoli were found in nine (52.9%), seven (41.1%), and six (35.2%) cases, respectively, while necrosis and calcification were occasionally observed. IHC staining results showed that CD31 and CD34 were predominantly positive, with ten (58.8%) and seven (41.1%) cases showing diffuse positivity, respectively. Factor VIII staining was diffusely positive in six (35.2%) cases. Ki-67 expression was negligible or low in six (35.2%) cases but moderate to high in three (17.6%) cases. Cytokeratin was negative in five (29.4%) cases, and two (11.7%) cases exhibited focal positivity (Table4).

### Pathological findings of myoid HE

Numerical and semi-quantitative parameters were also reviewed on each slide of myoid HE. A total of four cases were examined. The slides exhibited mild to moderate anaplasia, with three cases showing moderate sclerosis. All non-tumoral areas appeared normal, and the tumors had well-defined borders. Mitotic figures were observed in one case, but no atypical mitosis was found. Myoid HE presented with pleomorphism, intracytoplasmic vacuolization, eosinophilic cytoplasm, and spindling in all four cases (Figure-1-2-3). Most tumors displayed coarse chromatin, irregular nuclear borders, and prominent nucleoli. One case displayed micro-thrombus, but none of the cases exhibited necrosis, multiple nucleoli, vascular channels, or glomeruloid formation. IHC staining showed that the slides were often diffusely positive for CD34, CD31, and factor VIII antigen. The SMA marker was diffusely positive in all three cases that underwent IHC study, and CK was mostly negative, except in one case, which showed diffusely positive staining. Ki-67 labeling index was negligible or low positive in three cases (Table4).

### Pathological findings of kaposiform HE

We identified a singular case of kaposiform HE involving the spleen in a 59-year-old male patient who subsequently underwent total splenectomy. The tumor displayed a well-defined border and presented with mild anaplasia along with severe sclerosis with pleomorphism, intracytoplasmic vacuolization, eosinophilic cytoplasm, and spindling. Conversely, the non-tumoral spleen tissue exhibited normal characteristics. The tumor demonstrated diffuse positivity for markers such as CD31, CD34, factor VIII antigen, and SMA. The Ki-67 proliferation index was relatively low, at 5%, while CK staining yielded negative results for the tumor (Table4).

## Discussion

HE represents a group of rare, borderline malignant vascular tumors with diverse clinical presentations and diagnostic challenges. This study from Southern Iran provides a comprehensive clinico-pathologic analysis of 22 HE cases, offering valuable insights into its demographic, radiographic, and histological characteristics. The findings emphasize the importance of accurate pathological diagnosis and highlight surgical intervention as a highly effective treatment modality associated with significantly improved patient survival.

The results indicate that patients with liver tumors caused by HE typically presented with specific symptoms, particularly general abdominal pain or right upper abdominal pain, occurring in approximately 50% of all patients. In a review of 45 HE cases conducted by Shiba et al. [14], it was observed that the most common initial symptom was abdominal pain, with a prevalence of 18% among patients. This was followed by symptoms such as back pain, palpable tumors, weight loss, fatigue, cough, and epigastric pain. In addition to these symptoms, respiratory issues were also reported, including vocal paralysis, bronchial pneumonia, respiratory discomfort, and bloody sputum [9]. The results indicated that liver tumors typically appeared as hypo-dense masses with peripheral rim enhancement when observed through three-phasic enhanced CT scans. The data obtained from liver CT scans and ultrasonography were consistent with findings from other comprehensive reviews of epithelioid HE. Various studies have demonstrated that on CT scans, hepatic epithelioid HE tends to manifest as multiple low-attenuation nodules of varying sizes, primarily involving the subcapsular region and often leading to capsular retraction [15, 16].

In this study, microscopic analysis revealed consistent intracytoplasmic vacuolization and eosinophilic cytoplasm across all cases. Additionally, a majority of cases exhibited pleomorphism, coarse chromatin, irregular nuclei borders, prominent nucleoli, the formation of glomeruloid structures, and nuclear spindling. Studies have established that the distinctive features of epithelioid HE encompasses the arrangement of endothelial cells in nests and cords, the presence of spindle-shaped tumor cells, lumens of varying sizes that occasionally contain red blood cells, and cells containing intracytoplasmic vacuoles that can lead to the development of a signet-ring appearance [3, 17]. In this study, immunohistochemical staining for CD31 and CD34 predominantly yielded positive results, with ten cases showing diffuse positivity for CD31 and seven cases displaying diffuse positivity for CD34. A single study revealed that the diagnosis of hepatic epithelioid HE primarily relies on pathological examination, coupled with the use of various differential IHC markers such as CAMTA1, CD31, CD34, CD10, vimentin, and factor VIII antigen. As of now, there are no established standardized treatment protocols for HE, and surgical approaches, including curative resection and liver transplantation, continue to be the primary modes of treatment. Research findings have suggested that the presence of extra-hepatic metastasis may not necessarily preclude the consideration of resection or transplantation as treatment options [18].

The findings indicate that the most prominent symptoms associated with spleen involvement are a palpable mass and left upper quadrant pain. Patients displaying spleen involvement exhibited hypo-dense mass lesions on CT scans. Research has highlighted that in the advanced stages of the disease, severe symptoms may manifest, including gastric distention, nausea, vomiting, dyspnea, shoulder pain, and constipation [15, 19]. The results indicated that all four cases exhibited tumor characteristics such as pleomorphism, intracytoplasmic vacuolization, eosinophilic cytoplasm, and spindling. Additionally, most tumors displayed features such as coarse chromatin, irregular nuclei borders, prominent nucleoli, mild to moderate anaplasia, moderate sclerosis, and a well-defined tumor border. Studies have established that myoid HE or myoid angioendothelioma encompasses a spectrum of morphological attributes, ranging from epithelioid to spindle-shaped tumoral cells forming small vascular channels. Notably, myoid HE typically lacks necrosis, demonstrates low mitotic activity, and presents with mild atypia [20, 21]. We identified a singular case of kaposiform HE involving the spleen in a 59-year-old male patient who subsequently underwent total splenectomy. When kaposiform HE manifests in visceral organs, a range of symptoms may emerge, starting with non-specific symptoms in the early stages and progressing to organomegaly and the development of a palpable mass, with the specific symptoms varying depending on the tumor’s location [22]. In our study, the tumor displayed a well-defined border and presented with mild anaplasia along with severe sclerosis. Also, it revealed the presence of pleomorphism, intracytoplasmic vacuolization, eosinophilic cytoplasm, and nuclear spindling. Conversely, the non-tumoral spleen tissue exhibited normal characteristics. The tumor demonstrated diffuse positivity for markers such as CD31, CD34, factor VIII antigen, and SMA. The Ki-67 proliferation index was relatively low, at 5%, while CK staining yielded negative results for the tumor. In a study by Abdulrahman AA. et al., the neoplastic spindle cells were observed to disrupt the entire normal splenic structure by creating a “vague slit-like vascular space in a nodular growth pattern, separated by hypo-cellular hyalinized fibrous bands.” Additionally, microthrombi were identified within the glomeruloid capillary proliferation and in the enlarged vascular lumens. Notably, there were no significant findings of cytological atypia, mitotic activity, or marked nuclear pleomorphism in the examined specimens [23].

In the case of adult-type HE and considering the location of involvement, patients were classified based on their chosen treatment methods, which included surgical intervention (10 cases), chemotherapy (2 cases), and three patients who did not receive any therapy for various reasons. Overall, 4 (18%) patients unfortunately succumbed to the disease. Within the subgroup of patients who underwent surgical treatment, 90% were censored, indicating significantly better outcomes compared to the other treatment groups. Generally, the choice of the most appropriate treatment is contingent upon various factors, such as the patient’s overall health, tumor size, presence of metastasis, multi-organ involvement, and other influential variables [12]. In our medical center, surgical intervention yielded highly favorable results, albeit with a need for careful data interpretation. Other studies have indicated that, overall, liver transplantation has been suggested as the preferred treatment option for hepatic epithelioid HE due to its multicentric nature within the liver, consistent with the findings in the current study [13, 24].

This research possesses several key strengths that enhance the quality and validity of its findings.

HE is exceptionally rare, making large-scale studies nearly impossible. This paper provides a detailed, granular analysis of 22 cases, which is a significant number for this condition and offers crucial clinical insights that are difficult to obtain elsewhere. The study went beyond simple chart review. Its major strength lies in the detailed histopathological and IHC analysis. The paper provides a very clear picture of the demographic, clinical, radiological, and pathological profiles of HE in this population. It successfully differentiates between the behaviors of epithelioid, myoid, and kaposiform subtypes, which is essential for clinicians. A 10-year retrospective period (2009-2019) was used to identify cases, which is necessary to capture a sufficient number of patients with such a rare tumor. The study draws clear, clinically actionable conclusions. It provides strong evidence supporting surgical intervention as a highly effective treatment, which can directly inform patient management. In summary, the study’s strengths lie in its detailed, multi-faceted analysis of a rare tumor, providing a valuable reference for diagnosis, characterization, and treatment planning that will be highly useful for pathologists, surgeons, and oncologists who encounter this challenging disease. While this study provides valuable insights into HE, several important limitations must be considered. With only 22 patients, the study has limited statistical power. HE is a rare tumor, but this small cohort makes it difficult to draw definitive conclusions about prevalence, demographic associations, and the effectiveness of different treatment strategies. As a retrospective review from two affiliated hospitals in a single region of Iran, the study is subject to selection bias. The findings may not be generalizable to other populations or healthcare settings. The evaluation of treatments was observational, not randomized. Patients were not assigned to different treatments randomly; instead, treatments were chosen based on individual clinical circumstances. The paper does not specify a standardized follow-up duration for all patients. Without long-term, consistent follow-up, the true overall survival, recurrence rates, and long-term efficacy of treatments cannot be fully ascertained.

## Conclusion

This study provides a detailed clinico-pathologic evaluation of HE from Southern Iran, underscoring the critical relationship between tumor location and clinical presentation. The findings confirm that liver involvement primarily manifests as abdominal pain, while splenic tumors are associated with a palpable mass and left upper quadrant pain. Importantly, the research highlights that HE, though rare, can occur in pediatric and adults and must be included in the differential diagnosis of vascular lesions across all age groups. Pathologically, the distinct histological and immunohistochemical profiles of epithelioid and myoid HE subtypes were delineated, with features such as intracytoplasmic vacuolization, eosinophilic cytoplasm, and specific IHC markers (CD31, CD34) being instrumental in accurate diagnosis. From a therapeutic perspective, surgical intervention emerged as the most effective treatment modality, associated with a notably high survival rate of 90% among operated patients. These insights not only enhance the diagnostic precision for this rare tumor but also reinforce the value of surgical management in improving patient outcomes. Future multi-center studies with larger cohorts are recommended to further validate these findings and refine treatment protocols.

## Conflict of Interest

The authors declare there is no conflict of interest.

## AI Disclosure Statement

During the preparation of this manuscript, the authors used ChatGPT, OpenAI company for language editing, grammar improvement, and liboberry.com for reference management. After its use, the authors thoroughly reviewed, verified, and revised all AI-assisted content to ensure accuracy and originality. The authors take full responsibility for the integrity and final content of the published article.
